# Book Reviews

**Published:** 1900-04

**Authors:** 


A Practical Treatise on Medical Diagnosis. For the use of students and practitioners. By John H. Musser, M. D., Professor of Clinical Medicine, University of Pennsylvania, Philadelphia. New (third) edition, thoroughly revised. Octavo, 1082 pages, with 253 engravings and 48 full-page colored plates. Philadelphia: Lea Brothers & Co. 1900.

We might begin our notice of this book with a dissertation on the importance of diagnosis as a foundation to intelligent treatment, for Musser's treatise would furnish a splendid text for such a beginning. To do so, however, would be but to repeat what every student of medicine is taught, and what every sophomore ought to know. It would, therefore be a waste of valuable time to make more than a simple reference to this important fact.

This author divides his work into two parts, the first being devoted to the consideration of general diagnosis, and the second to special diagnosis. The first part is subdivided into general observations, the data to be obtained by inquiry, the data obtained by observation, the blood, and the morbid processes and their symptomatology. To general observation is given a chapter to data obtained by inquiry—two chapters, to data obtained by observation—eighteen chapters, to the blood and the morbid processes, etc.—one chapter each.

The second part, in which special diagnosis is considered, consist of eight chapters, treating of the nose and pharynx; diseases of the lungs and pleuræ; diseases of the heart, bloodvessels and mediastinum; diseases of the mouth, fauces, pharynx and esophagus; diseases of the stomach, intestines and peritoneum; diseases of the liver, spleen, and pancreas; diseases of the kidneys; and diseases of the nervous system.

From this synopsis it will be readily observed how comprehensive Musser's treatise is and how well he has classified it. When the first edition issued from the press it was easily discovered by an experienced person that it would become a rival of works on diagnosis already in the field, and it soon became a competitor for first place. This edition, largely rewritten, fairly plants it in the forefront. Schools have adopted it has a text-book, teachers are pleased with it and use it in their classes, students study it and from it learn the first and best principles, while practitioners consult it and are guided by it in the management of disease. When an author has accomplished all this in six years he has triumphed; the goal is reached.

A Treatise on the Principles and Practice of Gynecology. By E. C. Dudley, A. M., M. D., Professor of Gynecology in the Northwestern University Medical School, Chicago New (second) edition. In one very handsome 8vo volume of 717 pages, with 453 engravings, of which 47 are in colors, and eight colored plates. Philadelphia and New York: Lea Brothers & Co. 1900.

A reëxamination of this treatise in detail has confirmed our previously expressed opinion in the Journal (November, 1898,) as to its value. At that time we invited the attention of our readers to the somewhat unique lines upon which the work had been constructed, to the original material it contained, and to the excellent character of its illustrations.

In this second edition all of the excellent qualities of the first have been retained, because, almost without exception, they received the approbation of teachers, students, and reviewers. To these have been added a consideration of the functional disorders of menstruation and sterility, constituting four new chapters, that have been grouped to form part VI. The other five parts remain practically the same as to plan and material as in the first edition. Some of the chapters, however, for example those on cystitis and diagnosis, contain much new matter. The chapter on diagnosis is the best one we have ever read in a work on gynecology. Its arrangement is superb and its methods are unexcelled. It shows everywhere the handiwork of a masterworkman. The same might be said of many other parts of the treatise without exaggeration, but we wish to invite particular attention to the chapter on diagnosis, because of its excellence, and again because of the importance of the topic. It is so important always that the beginner should be duly impressed with the fact that diagnosis is the foundation stone of successful management.

In examining other parts and chapters it is ascertained that nearly every one has felt the effects of the reviser's hand. Words, phrases, sentences, and paragraphs have been expanded, reconstructed, or otherwise modified to add to their clearness, while new material has been introduced wherever necessary to keep the book abreast of the progress of the art. Besides all this, there are six full-page plates and thirty-one illustrations that were not contained in the former edition. The illustrations are of the best, many having been specially drawn for this treatise.

Professor Dudley is to be congratulated upon his most successful entrée into the arena of authorship. He is just the man to write a book, because he has something to say, says it well, and then stops. His book contains no padding—only practical material that is wanted by students of gynecology whether under- or post-graduates, teachers, professors or practitioners.

---

An American Text Book of Surgery. For practitioners and students. By Phineas S. Connor, M. D., Frederic S. Dennis, M. D., William W. Keen, M. D., Charles B. Nancrede, M. D., Roswell Park, M. D., Lewis S. Pilcher, M. D., Nicholas Senn, M. D., Francis J. Shepherd, M. D., Lewis A. Stimson, M. D., J. Collins Warren, M. D. and J. William White, M. D. Edited by William W. Keen, M. D., LL.D. and J. William White, M. D., Ph. D. Third edition. Thoroughly revised. Imperial 8vo, pp. xvi. 1228.—Price, $7 00 cloth; $8.00 sheep and $9.00 half-Russia. Philadelphia: W. B. Saunders, 925 Walnut Street. 1899.

The list of contributors above enumerated contains the names of some of the most distinguished surgeons in this country, hence the surprising success of this work is explained. The editors announce that it has been adopted as a text-book in more than 100 medical colleges, a circumstance that is more convincing as to the value of the book than would be any words that we could write.

The new subjects presented in this edition are (1) a full consideration of ortho-therapy; (2) leucocytosis; (3) post-operative insanity; (4) the use of dry heat at high temperatures; (5) Krönlein's method of locating the cerebral fissures; (6) Hoffa's and Lorenz's operations for congenital dislocations of the hip; (7) Allis's researches on dislocations of the hip-joint; (8) lumbar puncture; (9) the forcible reposition of the spine in Pott's disease; (10) the treatment of exophthalmic goiter; (11) the surgery of typhoid fever; (12) gastrectomy and other operations on the stomach; (13) several new methods of operating on the intestines; (14) the use of Kelly's rectal specula; (15) the surgery of the ureter; (16) Schleich's infiltration-method and the use of eucaine for local anesthesia; (17) Krause's method of skin-grafting; (18) the newer methods of disinfecting the hands; (19) the use of gloves, and a few others of minor importance.

In addition to this long list of new topics, several sections have been revised and enlarged, and a number of new illustrations have been introduced. The editors have thought best to cut out the chapters on the surgery of the eye and ear that appeared in the first two editions. This we think was wise because the surgery of those organs is so distinctly "special," that it is considered in works devoted to those topics. This book is sufficiently large without those chapters.

The last chapter in the treatise is devoted to the use of the *x*-ray in surgery. It is illustrated by two beautiful plates and contains the most recent information on the subject. We are of the opinion that this work will long continue to hold a most prominent place in the literature of American surgery. It fits the necessities of the teacher, pupil, and the every-day practitioner of surgery in the interior or country—sometimes called provincial—districts. Such an all-around treatise must necessarily present strong claims to the surgeon of today.

---

TREATISE ON ORTHOPEDIC SURGERY. By EDWARD H. BRADFORD, M. D., Surgeon to the Children's Hospital and the Samaritan Hospital; Assistant Professor of Orthopedic Surgery in Harvard Medical School; and Robert W. Lovett, M. D., Assistant Surgeon to the Children's Hospital; Surgeon to the Infants' Hospital. Illustrated with 621 engravings. Second revised edition. New York: William Wood & Company. 1899.

Bradford and Lovett have been recognised for some years as authority upon orthopedy, and when the first edition of this treatise appeared, they included therein diseases or deformities of the joints as belonging to the specialty of orthopedic surgery. This was in 1890, and later, when Young's treatise appeared (1894) he followed the same plan. It has now come to be adopted as a portion of the field legitimately attached to orthopedic literature and practice.

During the past ten years there has been much advance in this branch of the surgical art, that is to say, in the cure of deformities, no matter where they exist, but especially as involving the spine and the large joints. Deformities of the feet, too, are being studied more and more, so that gradually the unsightliness of crooked and flat-footed people is disappearing.

These authors have considered this latter subject in much detail and have presented it in the light of the latest scientific thought.

In the consideration of Pott's disease of the spine, Bradford and Lovett are exhaustive, both in pathology and treatment and have presented the latest and best methods of straightening crooked spines, concerning which there has been, in recent years, considerable change in methods, much of which is for the better. It has come to be possible, under the skilful application of the modern principles of orthopedic surgery to the young cripple, to cure or improve almost every deformity. This is one of the triumphs of surgery, not second to any other in its far-reaching results.

The illustrations in this treatise are numerous, practical, instructive and make even the novice quickly acquainted with the subjects they portray.

---

A TEXT-BOOK OF EMBRYOLOGY. FOR STUDENTS OF MEDICINE. By JOHN CLEMENT HEISLER, M. D., Professor of Anatomy in the Medico-Chirurgical College, Philadelphia. With 190 illustrations, 26 of them in colors. Philadelphia: W. B. Saunders, 925 Walnut street, 1899.

The study of embryology has acquired such great interest in connection with the teaching and proper comprehension of human anatomy in recent years that a demand has arisen for compact comprehensive works on this subject. The author as a teacher has been awake to this demand and has prepared a work sufficient to meet the demands and exigencies of the hour, and the result is a carefully prepared statement of the essentials of embryology.

The book is divided into eighteen chapters, chapter I. taking up the male and female sexual elements; maturation; ovulation; menstruation and fertilisation.

In chapter II. he deals with the segmentation of the ovum and formation of the blastodermic vesicle. In chapter III. is considered the germ layers and the primitive streak or the stage of the gastrula. Chapter IV. treats of the beginning differentiation of the embryo; the neural canal; the chorda dorsalis and the mesoblastic somites. Chapter V. describes the formation of the body wall of the intestinal canal, and of the fetal membranes. Chapter VI. treats of the decidua, the placenta and the umbilical cord. Chapter VII. deals with the development of the external form of the body. In the succeeding chapters the author treats of the development of the connective tissue of the body, and the lymphatic system; face and mouth cavity, vascular system, digestive system, respiratory system, genito-urinary system, nervous system, skin and appendages, sense organs, muscular system, and development of the skeleton and limbs.

A very instructive tabulated chronology of development follows, showing the extent of development at the end of each week up to the tenth week, and then of the months following, proving very helpful to those who desire to know the exact development at a certain period.

The book, on the whole, is an excellent one, and can be recommended as a text-book or work of reference to the busy practitioner. The illustrations are of a high order of merit, the full page colored plates being very elucidating and handsomely executed. Paper, binding and press-work are all first-class.

A Practical Treatise on Materia Medica and Therapeutics. By Roberts Bartholow, M. A., M. D., LL. D., Professor Emeritus of Materia Medica, General Therapeutics and Hygiene in the Jefferson Medical College of Philadelphia; formerly professor of Materia Medica and Therapeutics and of the Practice of Medicine in the Medical College of Ohio, etc., etc. Tenth edition, revised and enlarged. Octavo, pp. xxii.-866. New York: D. Appleton & Co. 1899.

Roberts Bartholow has been acknowledged so long an authority upon the subjects of which this book treats, that it seems almost a work of supererogation to print any critique or review of it; especially does this so appear when its tenth edition is offered for examination.

The first edition of Bartholow's treatise was published in 1876, when the literature on this subject was much sparser than now, particularly as regards American works. Stillé and H. C. Wood, it is true, had put forth excellent books and these together with Ringer's therapeutics, constituted the student's resources as well as the practitioner's stock. Bartholow, therefore, found a ready ear and a willing purse, for at once his book took position in the front rank, a place that it has continued to hold with ease and credit.

The ninth edition was sent out in 1896 and three years later this present edition was called for. Notwithstanding the fact that numerous authors are now in the field, this treatise continues to be listed as a text-book by the colleges, and is always popular with students and teachers. The author has kept it fresh by additions and changes, always taking up the newer remedies and discussing them with intelligence, and eliminating the obsolete as fast as they have become so. This has served to maintain uniformity in the size of the book through its several editions, which is convenient, not overgrown, nor yet too small; since it always has ample space for the discussion of all important details, and is yet not too bulky to be handled with ease.

The near approach of the decennial revision of the United States Pharmacopeia makes it important that all views of authors shall be well in hand regarding newer remedies and, in our opinion, those of Bartholow will carry great weight with the committee of revision.

---

Malsbary's Practice of Medicine. A Pocket Text-Book of Theory and Practice of Medicine. By George E. Malsbary, M. D., Assistant to the Chair of Theory and Practice of Medicine, Medical College of Ohio, Cincinnati. Just ready. In one handsome 12mo volume of 405 pages, with 45 illustrations. Cloth, $1 75 net. Philadelphia: Lea Brothers & Company. 1899.

This is a well arranged epitomé of the salient points in the practice of medicine. It contains six chapters, with the titles as follows: Infection, diseases of the organs of digestion, diseases of the organs of respiration, diseases of the organs of circulation, diseases of the blood, and diseases of the genito-urinary organs.

This is an excellent method of classification to be employed in a small work like this, for it makes it easy of reference, each head and sub-head being printed in display type. As a rule, condensations as related to internal medicine are of little avail, but this one has considerable merit. It is prepared in a simple, direct fashion, is devoid of word-padding, and will be found valuable as a prompter in exigencies, when time must be economised.

Tenth Annual Report of the State Commission in Lunacy, October 1, 1897, to September 30, 1898. Transmitted to the Legislature April 10, 1899. New York and Albany: Wynkoop-Hallenbeck-Crawford Co., State Printers. 1899. From Hon. Henry W. Hill, Member of Assembly, Buffalo.

This report is in the usual form of the state documents and contains important information concerning the administration of the hospitals for the insane. The president of the commission, Dr. Peter M. Wise, has had experience as superintendent of one of these hospitals, as well as long training as an assistant, hence brings practical knowledge to his duties in the commission. This document contains much that will interest alienists and neurologists, the material is well arranged, and its contents indicate improvement in the management of lunatics.

THE AMERICAN YEAR-BOOK MEDICINE OF AND SURGERY. Edited by GEORGE M. GOULD, M. D. In two handsome octavo volumes. Profusely illustrated. Cloth, $3.00 per volume, net; half morroco, $3 75 per volume, net. Philadelphia: W. B. Saunders, Publisher, 925 Walnut Street. 1900.

This annual summary of medicine and surgery is published in two volumes this year, because it is more convenient to handle in that form. We think this is a good plan for that reason as well as for another—namely, it enables one to purchase either or both volumes according to fancy or purse.

Some editorial changes have been made in several of the departments, but the general plan of the work remains unchanged. It is an excellent résumé of the literature of the year 1899, and will be found convenient as a book of reference by every busy physician. The price, too, is reasonable, while mechanically the book is faultless for a work of its class.

---

TRANSACTIONS OF THE RHODE ISLAND MEDICAL SOCIETY. Volume V. Parts III, IV and V. Providence: Snow & Farnham. 1896-97-98.

The transactions of this society are published in parts bound in paper. They contain much of interest from a scientific viewpoint, and deserve to be sent out in more enduring form. The society holds its meetings quarterly, hence four meetings are essential to create a "part." Beginning with 1900 the proceedings will be published in the journal of the society, lately established.

---

TRANSACTIONS OF THE STATE MEDICAL SOCIETY OF WISCONSIN, Vol. XXXIII., for the year 1899. Edited by CHARLES S. SHELDON, Secretary. Madison: Tracy Gibbs & Co. 1899.

This book contains the proceedings of the society had at Oshkosh, May 3, 4, and 5, 1899. The meeting was an interesting one and its business, executive and scientific, is well recorded. By vote at the previous meeting the professional papers were limited to fifty, with a view of expanding the discussions. But this limitation was found to be insufficient to accomplish the desired purpose, even with sessions beginning at 8 o'clock a. m., so, for the present year the papers are limited to thirty-five, in hopes to more satisfactorily meet the end in view. Under the new plan the program committee invites twenty-five members to read papers, and will accept ten volunteer communications selected in competition from the whole number offered. This is something unique in the conduct of State medical societies, and the result will be watched with interest.

---

TRANSACTIONS OF THE AMERICAN DERMATOLOGICAL ASSOCIATION at its Twenty-second Annual Meeting, held in Princeton, N. J., May 31 and June 1, and in New York City, June 2, 1899. JOHN T. BOWEN, M. D., Secretary. Concord, N. H.: The Rumford Press. 1899.

This excellent association is noted for the quality of its work, and is content to publish its annual volume in a very modest way. We are persuaded, however, that the scientific worth attached to its meetings would justify that gieater degree of permanency which boards and muslin would afford its publications. Paper covers do not find a ready place in one's library. At the meeting herein recorded, Dr. J. Nevins Hyde, of Chicago, served as president, and everything was done with accuracy and had scientific value. There are numerous half-tone illustrations that demonstrate interesting and rare conditions. The camera has aided dermatology quite as much as, if not more than, any specialty in medicine.

---

TRANSACTIONS OF THE MEDICAL SOCIETY OF THE STATE OF WEST VIRGINIA, held at Weston, May 17, 18 and 19, 1899. Wheeling: Daily Intelligencer Steam and Job Press. 1899.

This society was instituted in 1867. The modest volume of proceedings is worthy of a place among those of states of similar size and age. The president at this meeting was Dr. John L. Dickey, of Wheeling, and the secretary was Dr. G. A. Aschman, of the same city. Seventeen new members were admitted, and the next meeting will be held at Morgantown, the date of which does not appear in the book.

---

TRANSACTIONS OF THE WYOMING STATE MEDICAL SOCIETY. First Annual and Second Regular Meetings, held at Rawlins and Rock Springs, May and November, 1898. E. STUVER, M.D., Secretary. Denver: App Engraving and Printing Company. 1899.

These two brochures bound together contain the reports of the proceedings held at the first and second meetings of this society. The first meeting was for organisation and the report is devoid of scientific work. The second regular meeting was really the first annual meeting, and at this several papers of interest were read and discussed. It is an excellent plan for a society to publish its work from the first, no matter how meager it may be, for it will always then be found accessible for reference.

---

TRANSACTIONS OF THE OHIO STATE MEDICAL SOCIETY. Fifty-fourth Annual Meeting, held at Springfield, May 10–12, 1899. Edited by P. MAXWELL FOSHAY, M. D. Cleveland: J. B. Savage. 1899.

The medical profession in Ohio is a progressive body, a fact amply attested by the volume before us. It contains much of scientific interest and it is well put together. The president at this meeting, Dr. N. R. Coleman, of Columbus, chose for the subject of his address, The relation of medicine to the state. This is a timely topic, which was most admirably presented. Dr. Coleman is president of the Ohio State Board of Examination and Registration, hence is familiar with the subject from every viewpoint. He was chairman of the committee on minimum standards of the National Confederation of State Medical Examining Boards, and under his direction this committee last year made a most exhaustive report, published in the JOURNAL, August, 1899.

This excellent volume of society transactions is full of evidence that it was presided over by an officer of great executive capacity.

Transactions of the Texas State Medical Association. Thirty-first Annual Session, held at San Antonio, April 25, 26, 27 and 28, 1899. Edited by Hamilton A. West, M. D., Secretary. Austin: Von Boeckmann, Schutze & Co. 1899.

The State Medical Association of Texas has been noted for its good work for many years as well as for the excellent manner in which it is published; all of which testifies to the efficiency of its secretary. Furthermore, it gives strong evidence of the propriety of retaining the services of a good secretary as long as they can be secured. It is not easy to train a new one, and this post is one where experience counts for much.

The Texas State Medical Association is to be congratulated in having such a secretary, and upon its excellent book of transactions for the year 1899.

---

Transactions of the Medical Association of Central New York. Thirty-first annual meeting held at Auburn, N. Y, October 18, 1898. William C. Krauss, M. D., Editor. Volume V. Buffalo: Buffalo Medical Journal Print. 1899.

This progressive society presents its work in excellent form, which is an important point in the successful conduct of such a body. When a society allows its work to scatter hither and yon among the medical journals, or perhaps allows some of it to lapse without publication it loses a strong element of cohesive power. If these five volumes are now bound together, they will make a handsome book, which is well indexed in this number.

---

Eighteenth Annual Report of the State Board of Health of New York, for the year 1897. Transmitted to the legislature, February 15, 1898. In two volumes. Albany: Wynkoop-Hallenbeck-Crawford Co. 1898.

These volumes follow the lines usually laid down by a state health report. They contain the stereotyped "report" to the governor, which is a summary of the work of the board for the year, followed by a detailed recital of the specific application of that work, local, general, and technical.

We would suggest that it would be in good form instead of addressing the report to governor A or governor B, whose terms of office are comparatively short, and, as in the present instance, may have ceased before it is printed, that it be addressed "To the Governor of the State of New York," an office that is perpetual.

It would appear also,-that such an important document might discuss, with propriety, important sanitary problems, such for example, as the state care of the consumptive. In the period embraced by this document, it is stated that consumption destroyed 12,650 lives—more than 1,000 a month. Surely, some plan should be devised by an intelligent community to put a check to the ravages of such a frightful malady.

The second volume of the report contains the maps of the sewer system and sewage disposal works of the several villages that have been inspected during the year. The board is doing a work of vast benefit to the state, but is compelled to restrict its sphere on account of meager appropriations of money.

